# The Role of QuantiFERON-TB Gold Plus in Mycobacterium Tuberculosis Detection in a Severe HIV Immunocompromised Patient—Case Report

**DOI:** 10.3390/pathogens10111523

**Published:** 2021-11-21

**Authors:** Florentina Dumitrescu, Cătălina-Gabriela Pisoschi, Vlad Pădureanu, Andreea Cristina Stoian, Livia Dragonu, Lucian Giubelan

**Affiliations:** 1 Department of Infectious Disease, Faculty of Medicine, University of Medicine and Pharmacy of Craiova, 200349 Craiova, Romania; dumitrescu_florentina@yahoo.com (F.D.); andreea_plr@yahoo.com (A.C.S.); livia_dragonu@yahoo.com (L.D.); ligiubelan@yahoo.com (L.G.); 2 Department of Pharmaceutical Biochemistry, Faculty of Pharmacy, University of Medicine and Pharmacy of Craiova, 200349 Craiova, Romania; catalina.pisoschi@umfcv.ro; 3 Department of Internal Medicine, Faculty of Medicine, University of Medicine and Pharmacy of Craiova, 200349 Craiova, Romania

**Keywords:** HIV, tuberculosis, infection, QuantiFERON-TB

## Abstract

Tuberculosis (TB) is an important opportunistic infection in HIV-positive people. We are reporting a case of a 31-year-old HIV-infected patient who was hospitalized in July 2021 for dyspnea, cough with mucopurulent sputum and asthenia. He was confirmed to have Serratia liquefaciens pneumonia and acute respiratory failure. The evolution was unfavorable despite the antibiotic, pathogenic and symptomatic treatment. Because the patient had severe immunosuppression (CD4 count = 37 cell/mm^3^), we used QuantiFERON-TB Gold Plus for the detection of the Mycobacterium tuberculosis infection. The antituberculosis therapy was initiated, which resulted in a significant improvement of the general condition and the patient was discharged with the recommendation to continue antiretroviral therapy, antituberculosis treatment and Trimethoprim/Sulfamethoxazole—single tablet daily for the prophylaxis of Pneumocystis pneumonia.

## 1. Introduction

Tuberculosis (TB) is a major cause of mortality and morbidity among people living with human immunodeficiency virus (HIV) infection.

According to the World Health Organization (WHO), there were 10 million new TB cases in the world in 2018 and 2 million TB deaths, of which 1.2 million were reported among HIV-negative people and 251,000 among HIV-positive patients [1]. It is estimated that approximately a quarter of the world’s population have latent infection with Mycobacterium tuberculosis (MTB). Patients with latent tuberculosis infection (LTBI) have a 5–10% lifetime risk of developing active TB, and most cases occur within the first five years after infection [2].

Diagnosis and treatment of LTBI is the most effective strategy for controlling TB among HIV-positive patients [3]. The WHO recommended Isoniazid preventive therapy for people living with HIV, which reduced the risk of developing active TB by 62% [4].

The conversion from LTBI to active TB may be the result of significant disorders of the immune system. HIV infection is the most important risk factor for the reactivation of LTBI, TB remaining the leading cause of death among HIV-positive people. Patients co-infected with HIV and MTB have 26 to 31 times the increased risk of reactivation of LTBI [5]. Although antiretroviral therapy (ART) has reduced the incidence of TB among HIV-positive patients, TB rates remain high after the initiation of ART, and a large number of patients develop TB before being eligible to receive ART [6,7,8,9].

There are two diagnostic methods for detecting LTBI: the tuberculin skin test (TST) or Mantoux test and interferon-gamma release assays (IGRAs). 

A positive QFT result suggests that the person tested is likely to be infected with MTB. In this case, a medical evaluation based on the assessment of symptoms and risk factors, physical examination and a chest radiograph should be performed to establish the diagnosis of active TB or LTBI. 

## 2. Case Report

We present the case of a 31-year-old patient diagnosed with C3 category HIV infection in 2014, who interrupted antiretroviral therapy (ART) for about two years. He was admitted to the Emergency County Hospital from Târgu-Jiu in July 2021 for dyspnea, cough with mucopurulent sputum and asthenia. 

The objective examination at the time of admission revealed: severe general condition, underweight, pale skin and mucous membranes, poorly represented connective-adipose tissue, generalized micropolyadenopathy, muscle hypotonia and hypotrophy, bilateral crackles on chest auscultation, oxygen saturation = 93% with supplemental oxygen (5 L/min), normal heart sounds, tachycardia, heart rate = 130/min, blood pressure = 100/60 mmHg, white patches on the tongue, supple and painless abdomen, abdominal movement during breathing, no signs of meningeal irritation. Laboratory tests are presented in Table 1.

Throat swabs (fungal culture): *Candida albicans* (susceptible to Amphotericin B, Miconazole, Ketoconazole, Nystatin; resistance to Fluconazole)

RT-PCR SARS-CoV-2 nasopharyngeal swabs and sputum (three samples): negative 

Sputum samples (fungal culture): *Candida albicans* (susceptible *to* Amphotericin B, Miconazole, Ketoconazole, Nystatin; resistance to Fluconazole)

Bacteriological examination of sputum: *Serratia liquefaciens* (susceptible to Ceftazidime/Avibactam, Cefotaxime, Tetracycline, Ampicillin/Sulbactam, Meropenem, Trimethoprim/Sulfamethoxazole)

Sputum examination *MTB* (three samples): negative

Imaging exams

Chest X-ray: Thickening of the peribronchovascular interstitium localized in both the lungs and micronodules. Pleurodiaphragmatic adhesion in the oblique fissure in the left lung. Scissuritis and right periscissuritis.

**Figure 1:**
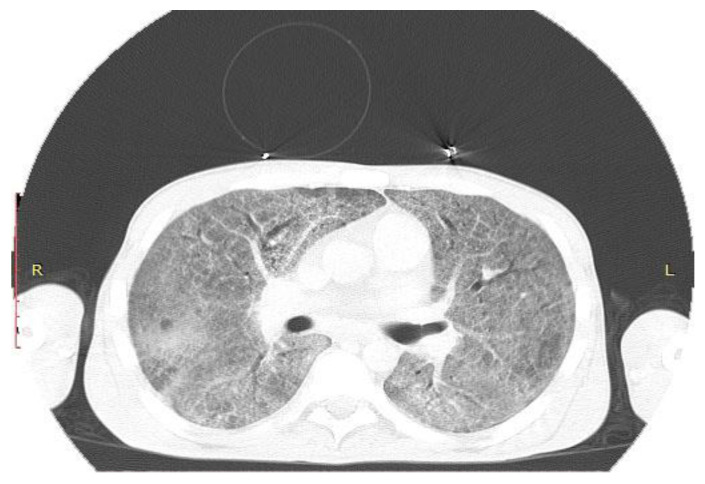
Aspects of pulmonary CT-ground-glass opacities. Repeated pulmonary CT scan (after three weeks) showing the same aspects.

CT scan of the chest: Ground-glass opacities of the same intensity in both lungs, except the lower segment; areas of hyperlucency in both lungs; mediastinal lymphadenopathy, with the size of 10 mm; no accumulation of pleural or pericardial fluids (Figure 1).

### Evolution and Treatment

During hospitalization, the patient received antibiotic treatment (Meropenem 3 g/day, Vancomycin 2 g/day, Moxifloxacin 400 mg/day), antifungals, bronchodilators, corticotherapy, oxygen therapy, albumin, oxygen therapy (7 days of high flow at intensive care unit), hepatoprotective, gastroprotective and symptomatic drugs. Antiretroviral therapy was initiated in August 2021 with Abacavir/Lamivudine + Efavirenz. After about one month of hospitalization (two of them with ART), the patient is transferred to the HIV/AIDS Department—“Victor Babeş” Clinical Hospital of Infectious Diseases and Pneumoftiziology Craiova.

At admission to the Victor Babes Hospital, the patient had dyspnea, cough, asthenia and diarrhea. Oxygen saturation was 95% with 10 L/min supplementary oxygen therapy, blood pressure 112/87 mmHg, sinus rhythm, ventricular allure = 110/min. Laboratory tests are presented in Table 2.

Sputum examination *MTB* (two samples, GeneXpert MT tests): negative;

Bacteriological examination of sputum: *Klebsiella pneumoniae* (susceptible to Colistin, resistance to Ampicilin, Cefotaxime, Cefoperazone/Sulbactam, Cefuroxime, Ceftriaxone, Ciprofloxacin, Meropenem);

Sputum examination: fungal culture = *Candida albicans* (susceptible to Miconazole, Econazole, Ketoconazole);

RT-PCR SARS- CoV-2 nasopharyngeal swab (two samples): negative; 

HIV viral load = 1251 copies/mL;

CD4 count = 37 cells/mm^3^;

*Clostridium difficile* Toxin A + B test: positive.

Because respiratory symptoms did not improve with antibiotic treatment (Colistin for Klebsiella pneumoniae, Trimethoprim/Sulfamethoxazole—high doses for potential *Pneumocystis jirovecii* pneumoniae, oral vancomycin for C.difficile colitis), nasal cannula oxygen therapy, corticotherapy, and the patient also presented fever, we completed the paraclinical investigation:

Blood cultures: negative;

Although the patient had several MTB negative sputum samples, we also took into consideration the LTB probability, so we did the tuberculin skin test.

The tuberculin skin test (TST): negative (0 mm);

Due to the severe immunosuppression, we also did the QFT-Plus test.

QFT-Plus = positive: Nil = 0.221 UI/mL (range ≤ 8 UI/mL), Mitogen = 9.556 UI/mL (range ≥ 0.5 UI/mL), TB1 = 4.384 UI/mL (range < 0.35 UI/mL), TB2 = 5 UI/mL (range < 0.35 UI/mL).

Because CT scanning was unavailable at that moment, we repeated the pulmonary X-ray: miliary aspect; 

Pulmonology consultation: It is highly recommended to start antituberculosis treatment, according to the following scheme: Isoniazid 300 mg + Rifampicin 450 mg + Pyrazinamide 1500 mg + Ethambutol 1200 mg, 7/7.

The patient underwent antibiotic treatment (Colistin, Vancomycin, Trimethoprim/Sulfamethoxazole), antifungal treatment (Nistatin), corticotherapy, ART, antituberculosis therapy, treatment with albumin, hepatoprotective and symptomatic drugs.

The patient was discharged aftertwo2 weeks of antituberculosis treatment, with improved general condition, without supplementary oxygen therapy indication, with the recommendation to continue ART, antituberculosis treatment and antibiotic treatment with Trimethoprim/Sulfamethoxazole—single tablet daily for the prophylaxis of *Pneumocystis* pneumonia. 

The final diagnosis for the patient was C3 category HIV infection, *Klebsiella pneumoniae* pneumonia, acute respiratory failure, Miliary tuberculosis, Oropharyngeal candidiasis and *Clostridium difficile* colitis.

## 3. Discussion

The diagnosis and treatment of LTBI are important steps in reducing the risk of progression to active disease.

TST is an in vivo test that measures the immune response to tuberculin-purified protein derivative (PPD), which contains a multitude of bacterial proteins, that are also present in the Bacillus Calmette-Guérin (BCG) vaccine. An induration ≥5 mm is considered positive for HIV-positive patients. Immunological reactions to the PPD reagent used in TST may be nonspecific and may increase the rates of false-positive results [10]. TST has many limitations: it uses a relatively nonspecific complex of antigens, and thus it can be false-positive in patients who have received a BCG vaccine or have been exposed to non-tuberculosis mycobacteria; it also may be false-negative in HIV-infected patients with low CD4 lymphocyte count [11].

The most commonly used interferon-gamma release assay in the detection of LTBI is QuantiFERON-TB Gold Plus (QFT-Plus) test, which appears to be more specific for MTB than TST. The test measures peripheral blood mononuclear cell release of interferon-gamma following stimulation of a mixture of synthetic peptides that simulates two specific MTB antigens: early secretory antigenic target (ESAT-6) and filtered culture protein 10 (CFP-10). QFT-Plus uses four collection tubes: Nile (negative control), mitogen (positive control), TB1 (contains peptides that stimulate CD4 + T cells) and TB2 (contains a cocktail of antigens consisting of ESAT-6 and CFP-10, to detect the release of interferon-gamma from CD8 + T cells as well as from CD4 + T cells). CD8 + T cells play an important role in the host immune response in controlling MTB infection. ESAT-6 and CFP-10 antigens are absent from all mycobacterial strains used in BCG vaccines and from most non-tuberculosis mycobacterial species, except *M. marinum, M. kansasii* and *M. szulgai* [12,13]. The test also includes a positive control, which allows distinction between valid results and anergic reactions [14].

Many different studies have analyzed the performance of QFT in people living with HIV. Some studies have shown that QFT sensitivity is impaired in HIV-positive patients compared to HIV-negative patients, but to a lesser degree than TST [6]. A significant benefit of QFT over TST is that it incorporates a positive control. Furthermore, like TST, QFT is based on the functionality of CD4 cells, and its performance may be negatively influenced by the low number of CD4 lymphocyte count in HIV-positive patients [15,16].

A study that was conducted in Spain, in 2012, on 373 HIV-infected patients with a median CD4 cell count of 470 cells/mm^3^ showed that interferon-gamma release assays were more sensitive than TST for diagnosis of LTBI. TCT, QFT and T-SPOT.TB were positive in 13.3%, 7.5% and 18.5% of cases, respectively. Those who did not have TB in their personal pathological history (277 patients) were tested. Twenty (7.2%) of the patients had a positive TST result. Simultaneous testing by QFT and TST showed a positive result for LTBI in 26 patients (8.6%). Testing with QFT and/or T-SPOT.TB showed 54 (17.9%) positive results for LTBI, and simultaneous testing with TCT, QFT and/or T-SPOT.TB showed a positive result for LTBI in 56 patients (18.5%) [3].

Another study that was performed in Barcelona, Spain, in 2011, evaluated the performance of QFT and TST for the detection of LTBI among HIV-positive patients. HIV-positive patients (n = 135) with a median CD4 lymphocyte count of 300 cells/mm^3^ and 135 controls were tested. HIV-positive patients who tested positive on either test received chemoprophylaxis. The prevalence of LTBI was 6.7% by TST and 9.6% by QFT among HIV-positive subjects, respectively, 34% by TST and 21.5% by QFT among the control group. TST reactivity decreased sharply as CD4 cells fell (15.8%, 10.3% and 0% for CD4 > 500, 301–500 and ≤ 300 cells/mm^3^). A less pronounced decrease occurred with QFT (15.8%, 13.8% and 0% for CD4 > 500, 301–500 and ≤100 cells/mm^3^) [17].

A cohort study conducted in Chennai, India, between April 2007 and March 2008, included 105 patients with HIV-TB coinfection, naive for ART and antituberculosis treatment. Of the 105 patients tested, QFT was positive in 65% of patients, negative in 18% of patients and indeterminate in 17% of patients. The sensitivity of the test was similar in pulmonary TB and extra-pulmonary TB patients. The positivity of the test was not influenced by low CD4 count, but some patients with CD4 < 200 cells/mm^3^ had indeterminate results. TST was performed in all 105 patients, with a sensitivity of 31%, which decreased as CD4 cell count fell [18].

Our patient had multiple opportunistic infections associated with HIV infection due to severe immunosuppression. In the context of the current COVID-19 pandemic, SARS-Co-V2 infection was also discussed, even if RT-PCR SARS-CoV-2 nasopharyngeal swabs were negative. 

Because we have a lot of patients with HIV-TB coinfection in our area, even our patient had negative sputum for MTB, we strongly suspected TB and looked for possible methods of diagnosis. TST was performed, but the result was negative, explicable by a low CD4 count. We continued with performing QFT.

Although several studies have shown that in HIV patients, QFT can be determined in case of severe immunosuppression, in this case, QFT turning positive proved to be the key factor in identifying the TB diagnosis. Most likely, it was all about LTB, and restarting ART led to an immune reconstitution inflammatory syndrome, with the onset of a miliary TB.

We consider that clinicians must insist on the confirmation or exclusion of TB, one of the main opportunistic infections in HIV-infected patients, especially in areas with a high incidence of bacillary infection.

## 4. Conclusions

Even in HIV-infected patients with severe immunosuppression, QFT Plus can be a useful test for diagnosing MTB infection. 

This case demonstrates the difficulty of diagnosing tuberculosis in a patient with severe immunosuppression due to HIV infection. The multiple associated respiratory infections and the negative results for MTB of the patient’s sputum were the main impediments. The QFT Plus proved to be very important in this case.

## Figures and Tables

**Table 1:** Tests at Tg Jiu Emergency Hospital.

Laboratory Test Report	Normal Range	at Admission	after 5 Days	after 10 Days	after 15 Days	after One Month
White blood cells	4.0–10.0 × 10^3^/mm^3^	6.4	9.0	15.9	6.9	4.0
Neutrophils	45–70%	85.2	94	94.8	97.3	94.2
Hemoglobin	13.11–17.2 g/dL	10.9	10.1	12.8	11.5	10.1
Hematocrit	40–50%	32,1	30.4	38.6	33.9	29.6
Mean corpuscular volume (MCV)	81–101 fL	84.8	85.2	87.2	87.9	87.2
Mean corpuscular hemoglobin (MCH)	27–35 pg	28.8	28.2	28.9	29.7	29.7
Platelets	150–450 × 10^3^/mm^3^	241	251	283	173	90
Erythrocyte sedimentation rate (ESR)	2–12 mm/h	70	50	30	50	25
Fibrinogen	200–450 mg/dL	577.3	374.2	529.4	521.1	163
C-Reactive Protein	0–5 mg/L	182.5	29.23	72.86	13.45	44.78
Transaminases (GPT, GOT)	0–50 U/L	GPT = 33 GOT = 72	GPT = 33 GOT = 54	GPT = 52 GOT = 40	GPT = 87 GOT = 79	GPT = 163 GOT = 103
Gamma GT	0–55 U/L	94	66	86	97	123
Serum creatinine	0.67–1.17 mg/dL	0.6	0.2	0.4	0.4	0.4
Serum ferritin	20–250 μg/L	694.81	734.84	709.04	1409.31	2591.31
Albumin	3.5–5.2 g/dL	2.1	2.30	2.75	2.8	2.37
Serum total protein	6.6–8.3 g/dL	5.8	5.4	6	5.5	4.4
Lactate dehydrogenase (LDH)	0–248 U/L	867	700	639	1092	1064

**Table 2:** Laboratory tests at Victor Babes Hospital from Craiova.

Laboratory Test Report	Normal Range	at Admission(“Victor Babeş” Clinical Hospital)	after 5 Days	at Discharge
White blood cells	4.0–9.0 × 10^3^/mm^3^	9.3	5.5	7.9
Neutrophils	34–71%	85	84.5	81.1
Hemoglobin	12.5–16 g/dL	12.1	9.7	9.7
Hematocrit	37–50%	36.8	29.1	29.9
Mean corpuscular volume (MCV)	75–95 μm^3^	93	92	93
Mean corpuscular hemoglobin (MCH)	26–32 pg	30.4	30.9	30
Platelets	150–450 ×10^3^/mm^3^	119	119	148
Erythrocyte sedimentation rate (ESR	1–12 mm/1 h 4–25 mm/2 h	35 mm/1 h 60 mm/2 h		25 mm/1 h 47 mm/2 h
Transaminases (GPT, GOT)	GPT = 10–35 U/L GOT = 0–32 U/L	GPT = 100 GOT = 73	GPT = 42 GOT = 48	GPT = 81 GOT = 67
Na^+^	136–145 mmol/L	132	128	128
K^+^	3.30–5.10 mmol/L	3.6	3.5	4.5
